# Phase 2 results of lisocabtagene maraleucel in Japanese patients with relapsed/refractory aggressive B‐cell non‐Hodgkin lymphoma

**DOI:** 10.1002/cam4.4820

**Published:** 2022-05-26

**Authors:** Shinichi Makita, Go Yamamoto, Dai Maruyama, Yuki Asano‐Mori, Daisuke Kaji, Revathi Ananthakrishnan, Ken Ogasawara, Lara Stepan, Claudia Schusterbauer, Nils Rettby, Jens Hasskarl, Koji Izutsu

**Affiliations:** ^1^ National Cancer Center Hospital Tokyo Japan; ^2^ Toranomon Hospital Tokyo Japan; ^3^ Bristol Myers Squibb Princeton New Jersey USA; ^4^ Celgene, a Bristol‐Myers Squibb Company Boudry Switzerland

**Keywords:** Japanese patients, large B‐cell lymphoma, lisocabtagene maraleucel, non‐Hodgkin lymphoma, relapsed/refractory

## Abstract

The autologous anti‐CD19 chimeric antigen receptor (CAR) T‐cell product, lisocabtagene maraleucel (liso‐cel), is administered at equal target doses of CD8^+^ and CD4^+^ CAR^+^ T cells. This analysis assessed safety and efficacy of liso‐cel in Japanese patients with relapsed or refractory (R/R) aggressive large B‐cell lymphoma (LBCL) in Cohort 3 of TRANSCEND WORLD (NCT03484702). Liso‐cel (100 × 10^6^ total CAR^+^ T cells) was administered 2–7 days after lymphodepletion. The primary efficacy endpoint was objective response rate (ORR; Lugano 2014 criteria) assessed by an independent review committee. Fourteen patients were enrolled; 10 received liso‐cel infusion (median time to liso‐cel availability, 23 days) and were evaluable at data cutoff (median follow‐up, 12.5 months). Grade ≥ 3 treatment‐emergent adverse events were neutropenia (90%), leukopenia (80%), anemia (70%), and thrombocytopenia (70%). All‐grade cytokine release syndrome (CRS) was observed in 50% of patients, though no grade ≥3 CRS events were reported. Grade 1 neurological events occurred in 1 patient but were resolved without any intervention. Prolonged cytopenia (grade ≥ 3 at day 29) was reported for 60% of patients. The ORR was 70%, and complete response rate was 50%. The median duration of response was 9.1 months (95% confidence interval [CI], 2.1—not reached), and overall survival was 14.7 months (95% CI, 1.7—not reached). One patient diagnosed with central nervous system involvement after screening but before liso‐cel infusion, responded to liso‐cel. Liso‐cel demonstrated meaningful efficacy and a manageable safety profile in Japanese patients with R/R LBCL.

## Graphical abstract

This analysis assessed safety and efficacy of lisocabtagene maraleucel (liso‐cel), an autologous CD‐19‐directed CAR T‐cell product, in Japanese patients with R/R aggressive LBCL in Cohort 3 of TRANSCEND WORLD (NCT03484702). Liso‐cel demonstrated meaningful efficacy and a manageable safety profile in Japanese patients with R/R LBCL. We also report the first case of a patient who was diagnosed with secondary CNS lymphoma and achieved a response following treatment with liso‐cel.

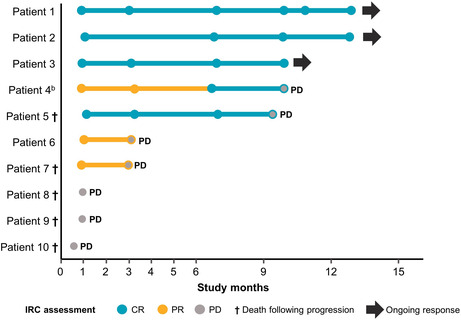

## 1 INTRODUCTION

Diffuse large B‐cell lymphoma (DLBCL) accounts for one‐third of all non‐Hodgkin lymphomas (NHL) in Japan.^1^, ^2^ Although the introduction of the anti‐CD20 monoclonal antibody rituximab has improved the prognosis of patients with DLBCL in recent decades, nearly 40% of patients experience relapsed and/or refractory (R/R) disease after rituximab‐containing first‐line chemotherapy.^3^ Salvage cytotoxic chemotherapy followed by high‐dose chemotherapy with autologous hematopoietic stem cell transplantation (auto‐HSCT) is the standard of care in patients with first relapse or refractory (R/R) DLBCL. However, this strategy is limited to relatively younger patients (usually 65 years or younger) with adequate organ function and chemotherapy‐sensitive disease. Once the disease becomes refractory, it is difficult to manage it with conventional cytotoxic chemotherapies. A multicohort retrospective study of outcomes in North America and Europe reported a median overall survival (OS) of 6.3 months in patients with refractory DLBCL^4^; similar outcomes (7 months) have been reported in the Japanese population.^5^

Several novel agents have been developed for patients with R/R large B‐cell lymphoma (LBCL), including DLBCL. Among them, anti‐CD19 chimeric antigen receptor (CAR) T‐cell therapies are considered one of the most promising treatment options for this population.^6^, ^7^, ^8^, ^9^, ^10^, ^11^, ^12^, ^13^, ^14^, ^15^ The CD19‐directed autologous CAR T‐cell product, lisocabtagene maraleucel (liso‐cel), is administered as a sequential infusion of equal target doses of CD8^+^ and CD4^+^ CAR^+^ T cells. Liso‐cel production involves purifying CD8^+^ and CD4^+^ cells separately before activation and transduction followed by T‐cell‐specific activation and expansion, which reduces the variability of CD3 T‐cell frequencies, relative to leukapheresis.^16^, ^17^, ^18^

In animal models, a defined composition of CD8^+^ and CD4^+^ CAR^+^ T cells demonstrated better expansion and was associated with improved efficacy compared with treatment using either T‐cell component separately.^19^ Administering a defined composition of CD8^+^ and CD4^+^ CAR^+^ T cells results in a more uniform CAR T‐cell product and may allow the identification of factors associated with CAR T‐cell kinetics and outcomes.^20^ Liso‐cel consists of autologous T cells transduced with a lentiviral vector encoding a CAR that incorporates an anti‐CD19 single‐chain variable fragment, a 4‐1BB costimulatory motif, and a CD3ζ T‐cell activation domain.^21^, ^22^ Growing evidence from preclinical studies suggests that the 4‐1BB costimulatory domain may enhance the persistence and safety of CAR T‐cell therapy.^23^, ^24^

Liso‐cel safety and efficacy were assessed in the multicenter, multicohort, seamless design, pivotal United States study, TRANSCEND NHL 001 (NCT02631044). In that study, liso‐cel demonstrated rapid and durable responses in patients with R/R LBCL.^6^ The objective of this study (TRANSCEND WORLD [NCT03484702]) was to characterize the safety and efficacy of liso‐cel treatment in non‐US patients with R/R LBCL; here we report the results of Cohort 3, which only enrolled patients in Japan.

## 2 MATERIALS AND METHODS

### 2.1 Study design and patient population

TRANSCEND WORLD is a single‐arm, open‐label, multinational, multicohort, phase 2 study (Appendix S1). Results from Cohort 3 are reported here and follow‐up in this cohort is ongoing.

Adult Japanese patients who met the following criteria were eligible for the study: R/R LBCL (DLBCL de novo or transformed follicular lymphoma, high‐grade B‐cell lymphoma with *MYC* and *BCL2* and/or *BCL6* rearrangements with DLBCL histology, or follicular lymphoma grade 3B), adequate organ function, and either receipt of ≥2 prior lines of therapy and Eastern Cooperative Oncology Group performance status (ECOG PS) score ≤ 1 or receipt of 1 prior line of therapy and not eligible for transplantation with ECOG PS ≤2. Prior treatment with an anti‐CD20 antibody and anthracycline was required. Secondary central nervous system (CNS) lymphoma was allowed.

We adhered to the ethical principles of the Declaration of Helsinki and Good Clinical Practice guidelines. Before screening, written informed consent was obtained from each patient. An independent ethics committee at each site reviewed and approved the protocol before initiating the study.

Patients underwent leukapheresis to collect autologous peripheral blood mononuclear cells for the manufacture of liso‐cel. Target processing blood volume was determined by the number of peripheral blood lymphocytes. Patients with a lymphocyte count ≥500/μL had processing volumes of 7 L, while patients with lymphocytes <500/μL had processing volumes of 12 L. After leukapheresis, fresh leukapheresate was shipped to the manufacturing site without cryopreservation. All CAR T‐cell products in this study were manufactured at the sponsor's manufacturing facility in Bothell, WA, USA. Leukapheresis material was immunomagnetically selected for CD8^+^ and CD4^+^ T cells, which were then independently activated, transduced with the liso‐cel CAR construct, and expanded in order to obtain the cell numbers required for infusion at the target doses of CD8^+^ and CD4^+^ CAR^+^ T cells. Both the CD8^+^ and CD4^+^ CAR^+^ T‐cell components were required to meet quality specifications prior to release for infusion and to be considered liso‐cel. However, patients could receive a nonconforming CAR T‐cell product (i.e., one of the CD8 or CD4 cell components did not meet one of the requirements to be considered liso‐cel).

Bridging chemotherapy (systemic and/or radiation) was allowed at the discretion of the treating clinician during the liso‐cel manufacturing period. Patients had to continue to meet eligibility criteria for lymphodepleting chemotherapy (LDC)/infusion, including having PET‐positive disease before proceeding to study treatment.

LDC comprising fludarabine (30 mg/m^2^) and cyclophosphamide (300 mg/m^2^) was administered intravenously daily for 3 days once the liso‐cel product had become available. Liso‐cel (target dose of 100 × 10^6^ total CAR^+^ T cells) was administered 2–7 days after LDC.

### 2.2 Study assessments

The primary endpoint was the objective response rate (ORR, defined as the proportion of patients achieving a complete response [CR] or partial response [PR]) as assessed by an independent review committee (IRC) per the Lugano 2014 PET‐computed tomography (CT) criteria^25^; secondary endpoints included safety, CR rate, duration of response (DOR), progression‐free survival (PFS), and OS. The cellular kinetic profile, incidence of B‐cell aplasia, and laboratory abnormalities were also explored. DOR was defined as the time from first response (CR or PR) to disease progression or death. PFS was defined as the time from liso‐cel infusion to disease progression or death. OS was defined as the time from liso‐cel infusion to death. Adverse events (AE), laboratory abnormalities, vital signs, and other safety measures were collected at protocol‐defined time points. AEs were graded using National Cancer Institute Common Terminology Criteria for Adverse Events (NCI CTCAE) v4.03. Neurological events (NE) were defined as investigator‐identified neurological AEs related to liso‐cel and were graded using NCI CTCAE v4.03. Lee 2014^26^ criteria were used to grade cytokine release syndrome (CRS). Investigators were trained in the recognition, management, and grading of CRS and NEs, including the importance of excluding other causes of fever or neurological symptoms. Suspected cases of CRS and NEs were managed according to the recommended treatment algorithms (Appendix S1). Treatment of CRS included tocilizumab (not to exceed 3 doses in 24 h and 4 total doses) and increasing dexamethasone as indicated. Treatment of NE included dexamethasone, with dose and frequency adjusted on grade and response to treatment. Recommendations for the highest‐grade event were used if concurrent CRS and NE were present. Prolonged cytopenia was defined as laboratory‐assessed neutropenia, thrombocytopenia, or anemia of grade ≥ 3 not resolved by study day 29. Cellular kinetics, based on quantitative polymerase chain reaction, were assessed as described previously.^6^, ^27^ B‐cell aplasia (defined as <3% CD19^+^ peripheral blood lymphocytes) was analyzed by T‐cell, B‐cell, and natural killer cell detection assay.

### 2.3 Statistical analysis

This primary efficacy analysis for Cohort 3 was planned to initiate after 10 patients were treated with liso‐cel and followed for at least 3 months. Statistical analyses were based on the following analysis sets: liso‐cel‐treated set (all patients who received liso‐cel; primary efficacy and safety analysis set), efficacy‐evaluable set (all patients who received liso‐cel, had baseline assessment and with ≥1 valid post‐liso‐cel disease assessment), and leukapheresed set (all patients who underwent leukapheresis). For binary endpoints, the frequency distribution (n, %) and two‐sided exact 95% confidence intervals (CI) were calculated. ORR was calculated with a two‐sided 95% exact Clopper–Pearson CI. Time‐to‐event endpoints, such as PFS, utilized Kaplan–Meier product limit methodology to estimate the survivorship function, and medians with a two‐sided 95% CI were calculated.

### 2.4 Data sharing statement

Bristol Myers Squibb policy on data sharing may be found at https://www.bms.com/researchers‐and‐partners/independent‐research/data‐sharing‐request‐process.html.

## 3 RESULTS

### 3.1 Patient demographics

**FIGURE 1:**
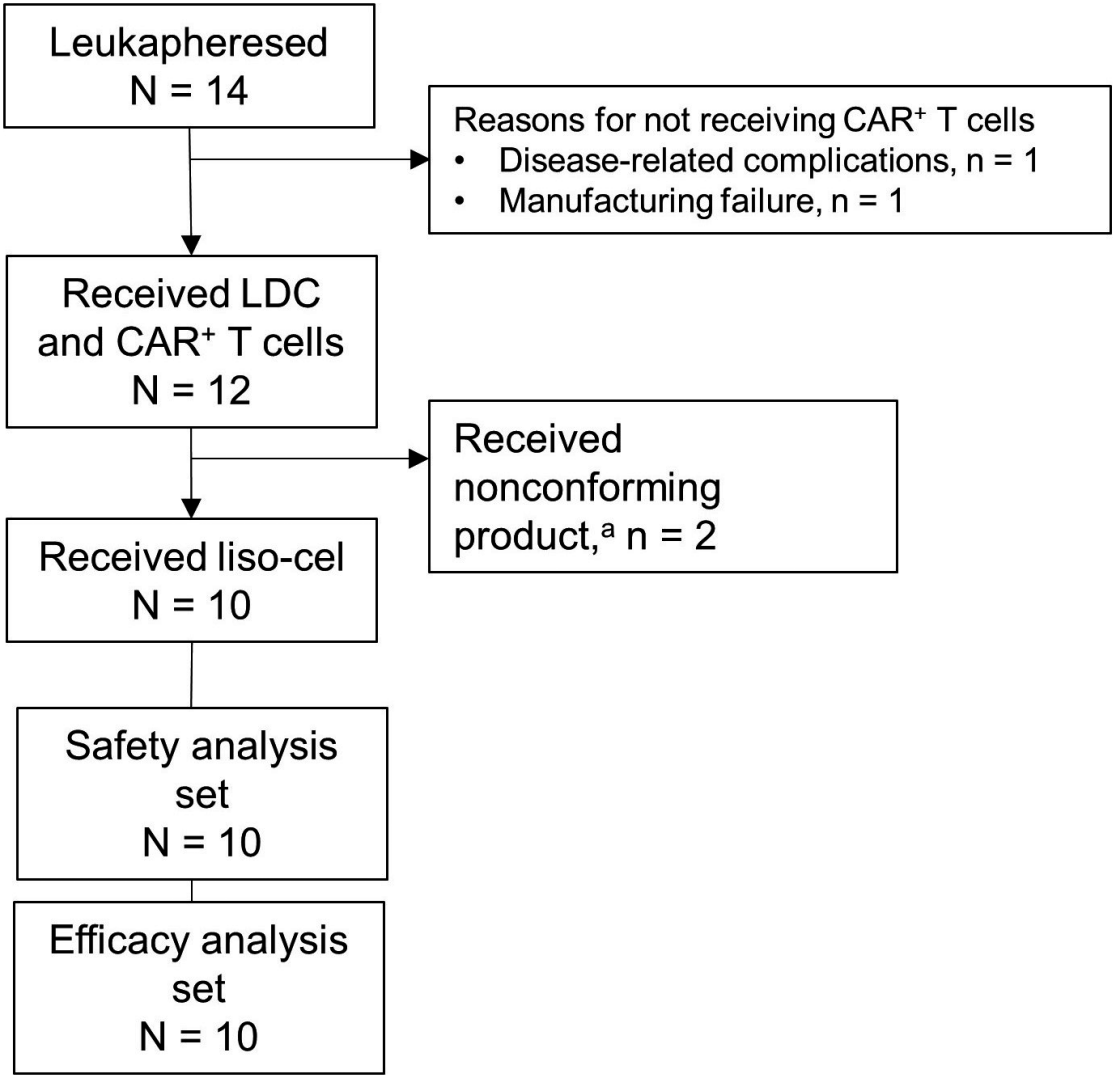
Patient flow. ^a^Liso‐cel consists of equal target doses of CD8^+^ and CD4^+^ CAR^+^ T cells, each of which was required to meet quality specifications. Although CAR T cells could be manufactured for all but one patient, the product for two patients did not meet the specifications of liso‐cel (i.e., 1 of the CD8^+^ or CD4^+^ cell components did not meet one of the requirements to be considered liso‐cel). CAR, chimeric antigen receptor; LDC, lymphodepleting chemotherapy; liso‐cel, lisocabtagene maraleucel

As of the June 19, 2020, data cutoff for this primary analysis, 14 patients in Cohort 3 underwent leukapheresis for the manufacture of CAR^+^ T cells (Figure 1). Two patients did not receive CAR^+^ T cells: 1 was no longer eligible for treatment because of deep vein thrombosis and 1 because of a manufacturing failure. Two patients received a nonconforming CAR^+^ T‐cell product (i.e., release specification criteria for liso‐cel not met, but product considered safe for infusion). For the 10 patients who received liso‐cel, the median turnaround time (time from leukapheresis to product availability) was 23 (range, 21–33) days. The median study follow‐up for the 10 patients who received liso‐cel infusion and were evaluable for both safety and efficacy at the data cutoff was 12.5 months. Follow‐up is ongoing.

Table 1 shows characteristics at baseline of the 10 patients who received liso‐cel. Median age was 57 (range, 47–73) years and 3 patients were aged ≥65 years. Patients were heavily pretreated, with a median of 3 (range, 1–9) prior lines of systemic therapy; 7 patients had refractory disease (defined as less than a CR to last therapy) and 3 had relapsed after initially responding to their last prior therapy. Two patients had received prior auto‐HSCT. Bridging therapy was administered to all 10 patients at the investigator's discretion (Table S1). The histologic subgroups included DLBCL not otherwise specified (*n* = 9, of which *n* = 3 had transformed follicular lymphoma) and follicular lymphoma grade 3B (*n* = 1). This cohort included the first report of a patient in Japan with DLBCL and secondary CNS lymphoma (diagnosed after screening) treated with CAR T‐cell therapy.

**TABLE 1:** Baseline patient and disease characteristics

Characteristic	Liso‐cel‐treated patients (*N* = 10)
Age, year	
Median (range)	57 (47–73)
≥65, *n* (%)	3 (30)
Male, *n* (%)	6 (60)
NHL subtype, *n* (%)	
DLBCL NOS	9 (90)
tFL	3 (30)
HGBCL	0
FL3B	1 (10)
Cell of origin, *n* (%)	
GCB	4 (40)
non‐GCB	5 (50)
N/A	1 (10)
Confirmed secondary CNS lymphoma at screening, *n* (%)	0
ECOG PS score before LDC, *n* (%)	
0	6 (60)
1	4 (40)
Prior therapy, *n* (%)	
Prior lines of therapy, median (range), n	3 (1–9)
Received prior HSCT	2 (20)
Refractory to last therapy	7 (70)
Relapsed after last therapy	3 (30)
LDH ≥500 U/L before LDC, *n* (%)	2 (20)
SPD ≥50 cm^2^ before LDC, *n* (%)	2 (20)
Received bridging therapy, *n* (%)	10 (100)

Abbreviations: CAR, chimeric antigen receptor; CNS, central nervous system; DLBCL, diffuse large B‐cell lymphoma; ECOG PS, Eastern Cooperative Oncology Group performance status; FL3B, follicular lymphoma grade 3B; GCB, germinal center B cell; HGBCL, high‐grade B‐cell lymphoma; HSCT, hematopoietic stem cell transplantation; LDC, lymphodepleting chemotherapy; LDH, lactate dehydrogenase; liso‐cel, lisocabtagene maraleucel; N/A, not applicable; NHL, non‐Hodgkin lymphoma; NOS, not otherwise specified; SPD, sum of the product of perpendicular diameters; tFL, transformed follicular lymphoma.

### 3.2 Safety

The most common any‐grade treatment‐emergent AEs (TEAE) were neutropenia (90%), thrombocytopenia (90%), leukopenia (90%), anemia (80%), CRS (50%), fatigue (40%), hypofibrinogenemia (40%), and hypogammaglobulinemia (20%; all were grade 1 or 2). Grade ≥ 3 TEAEs were neutropenia (90%), leukopenia (80%), anemia (70%), and thrombocytopenia (70%) (Table 2). No other severe TEAEs were reported. Severe thrombocytopenia generally began at post‐infusion day 15 (Figure S1). One patient, who had very rapid progressive disease, experienced a grade 5 TEAE of multiple organ dysfunction syndrome that was deemed not related to liso‐cel. Among the 10 patients, 5 experienced any‐grade CRS (3 grade 1; 2 grade 2) and 1 developed grade 1 NEs. No grade ≥3 CRS or NEs were reported (Table 3). Of the 4 patients with reported hypofibrinogenemia, 3 experienced grade 1 or 2 CRS and one patient had a grade 2 NE. One patient experienced hemorrhage (grade 2) after a needle biopsy and one patient received a plasma transfusion. No severe bleeding events were reported.

**TABLE 2:** Patient incidence of TEAEs

Parameter	Liso‐cel‐treated patients (*N* = 10)
Patients with TEAEs, *n* (%)	
Any TEAE	10 (100)
Grade 3	1 (10)
Grade 4	7 (70)
Grade 5^a^	1 (10)
Any liso‐cel‐related TEAE, n (%)	8 (80)
Grade 3	2 (20)
Grade 4	5 (50)
Grade 5	0
Grade 3/4 TEAEs, n (%)	
Neutropenia	9 (90)
Leukopenia	8 (80)
Anemia	7 (70)
Thrombocytopenia	7 (70)

Abbreviations: Liso‐cel, lisocabtagene maraleucel; TEAE, treatment‐emergent adverse event.

^a^ Multiple organ dysfunction syndrome caused by disease progression.

**TABLE 3:** AEs of special interest and their management

Parameter	Liso‐cel‐treated patients (*N* = 10)
Patients with TEAEs, *n* (%)	
CRS (Lee 2014 criteria),^26^	
All grades, *n* (%)	5 (50)
Grade 1	3 (30)
Grade 2	2 (20)
Time to onset, median (range), days	3 (2–9)
Time to resolution, median (range), days	4 (1–5)
Grade ≥ 3 CRS *n* (%)	0
NEs	
All grades, *n* (%)	1 (10)
Grade 1	1 (10)
Time to onset, days	4
Time to resolution, days	3
Grade ≥ 3, *n* (%)	0
Management of CRS and/or NEs, *n* (%)	
Tocilizumab only for CRS management	2 (20)
Corticosteroids only for NEs management	0
Tocilizumab and corticosteroids for concurrent CRS and NEs	1 (10)
Prolonged cytopenia,^a^ *n* (%)	6 (60)

Abbreviations: AE, adverse event; CRS, cytokine release syndrome; liso‐cel, lisocabtagene maraleucel; NE, neurological event; TEAE, treatment‐emergent adverse event.

^a^ Based on laboratory assessments of neutropenia, thrombocytopenia, or anemia of grade ≥3 not resolved by study day 29.

Median times to CRS onset and resolution were 3 (range, 2–9) and 4 (range 1–5) days, respectively. In the patient who developed NEs, time to NE onset and resolution were 4 days and 3 days, respectively. Two patients received tocilizumab for CRS management (1 grade 1, and 1 grade 2); 1 patient experienced CRS (grade 2) and NEs (grade 1) and received tocilizumab and corticosteroids for symptom management. Prolonged cytopenia was reported in 60% of patients. One patient (10%) experienced a grade 2 infection.

### 3.3 Efficacy

**FIGURE 2:**
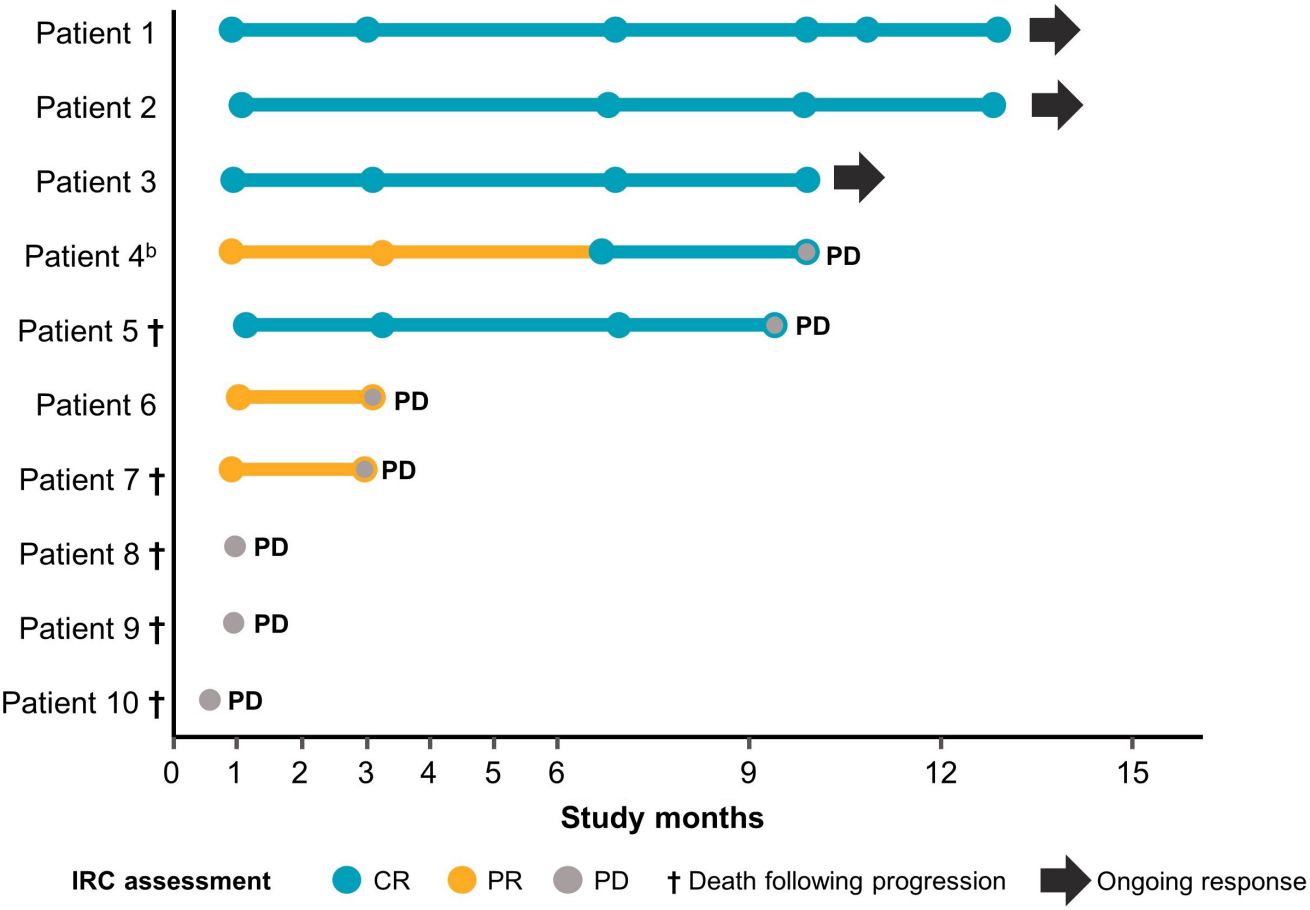
Primary analysis of individual patient responses to liso‐cel over time. ^a^The first dot represents the actual assessment date on or around day 29. ^b^One patient was diagnosed with secondary CNS lymphoma after screening and achieved a CR as a best overall response and was alive at the last assessment. CNS, central nervous system; CR, complete response; IRC, independent review committee; liso‐cel, lisocabtagene maraleucel; PD, progressive disease; PR, partial response

All 10 liso‐cel‐treated patients were evaluable for response and were included in the efficacy evaluations. The ORR by IRC was 70%. CR and PR rates were 50% and 20%, respectively (Table 4). Four patients achieved a CR on day 29; 1 patient experienced a PR on day 29, which later deepened to a CR. Three patients did not respond to liso‐cel and had progressive disease. The median DOR was 9.1 months (95% CI, 2.1—not reached [NR]), median PFS was 6.3 months (95% CI, 0.6—NR), and median OS was 14.7 months (95% CI, 1.7—NR). Of the 5 patients who achieved a best overall response of CR, 3 continued to maintain this response at the data cutoff date (Figure 2). A total of 5 liso‐cel‐treated patients have died due to disease progression and/or medical complications considered unrelated to liso‐cel treatment.

**TABLE 4:** Summary of efficacy responses

IRC‐assessed response (Lugano 2014 criteria)^25^	Liso‐cel‐treated patients (*N* = 10)
ORR, *n* (%)	7 (70)
CR	5 (50)
PR	2 (20)
SD	0
PD	3 (30)
DOR, median (95% CI), months	9.1 (2.1—NR)
PFS, median (95% CI), months	6.3 (0.6—NR)
OS, median (95% CI), months	14.7 (1.7—NR)

Abbreviations: CI, confidence interval; CR, complete response; DOR, duration of response; IRC, independent review committee; liso‐cel, lisocabtagene maraleucel; NR, not reached; ORR, objective response rate; OS, overall survival; PD, progressive disease; PFS, progression‐free survival; PR, partial response; SD, stable disease.

### 3.4 Cellular kinetics

**FIGURE 3:**
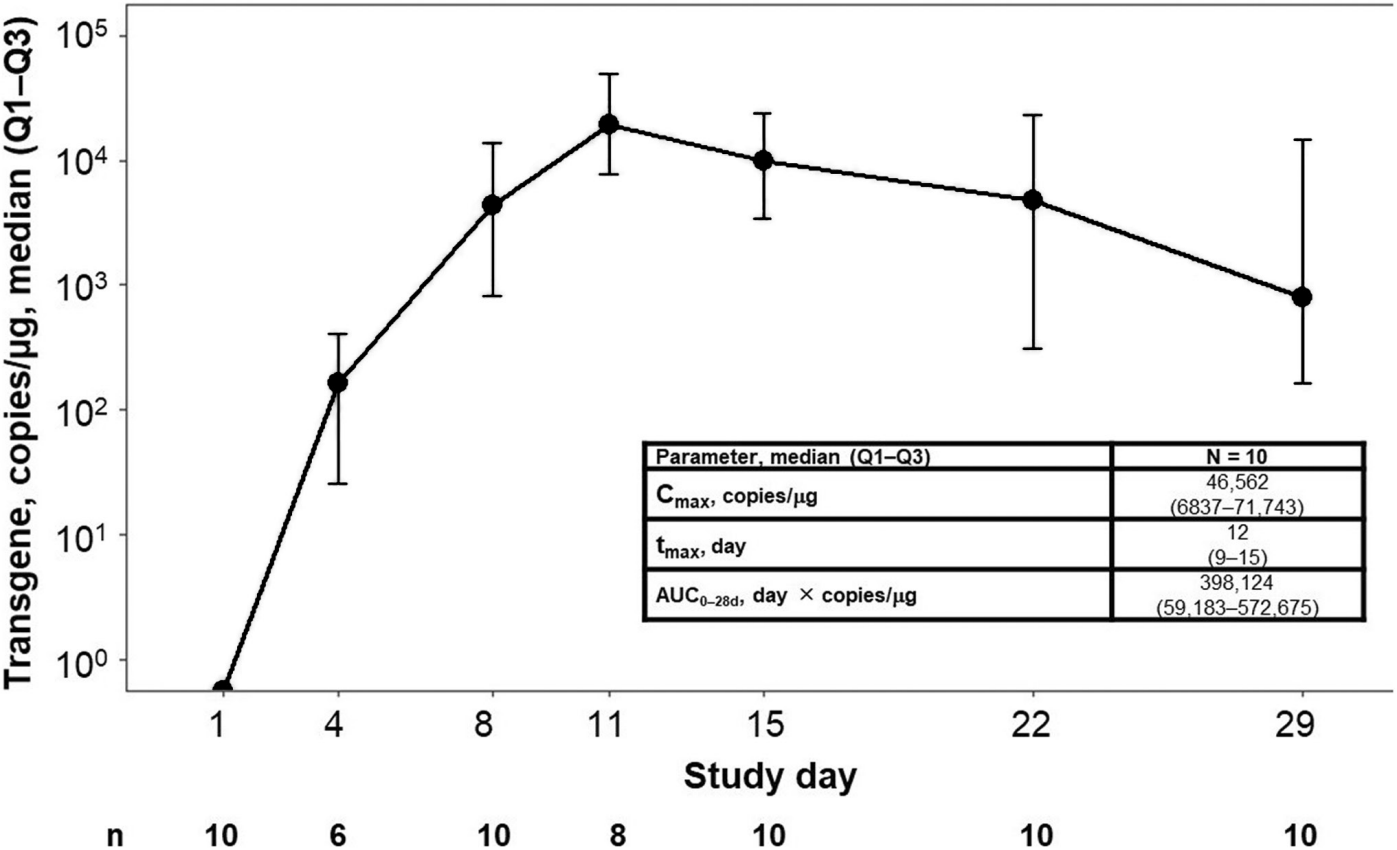
Cellular expansion over time. AUC_0–28d_, area under the curve from 0 to 28 days post‐infusion; C_max_, maximum expansion; Q, quartile; t_max_, time to C_max_

Among 10 patients evaluable for cellular kinetic analyses, median time to CAR T‐cell peak expansion was 12 days, median maximum expansion was 46,562 copies/μg, and median area under the curve from 0 to 28 days post‐infusion was 398,124 day × copies/μg (Figure 3). There were no apparent differences in cellular kinetics observed between responders and nonresponders (Figure S2). CAR T cells showed long‐term persistence in 71% of evaluable patients (*n* = 5/7) at 6 months and in 100% of evaluable patients (*n* = 2/2) at 1 year.

### 3.5 B‐cell aplasia

**FIGURE 4:**
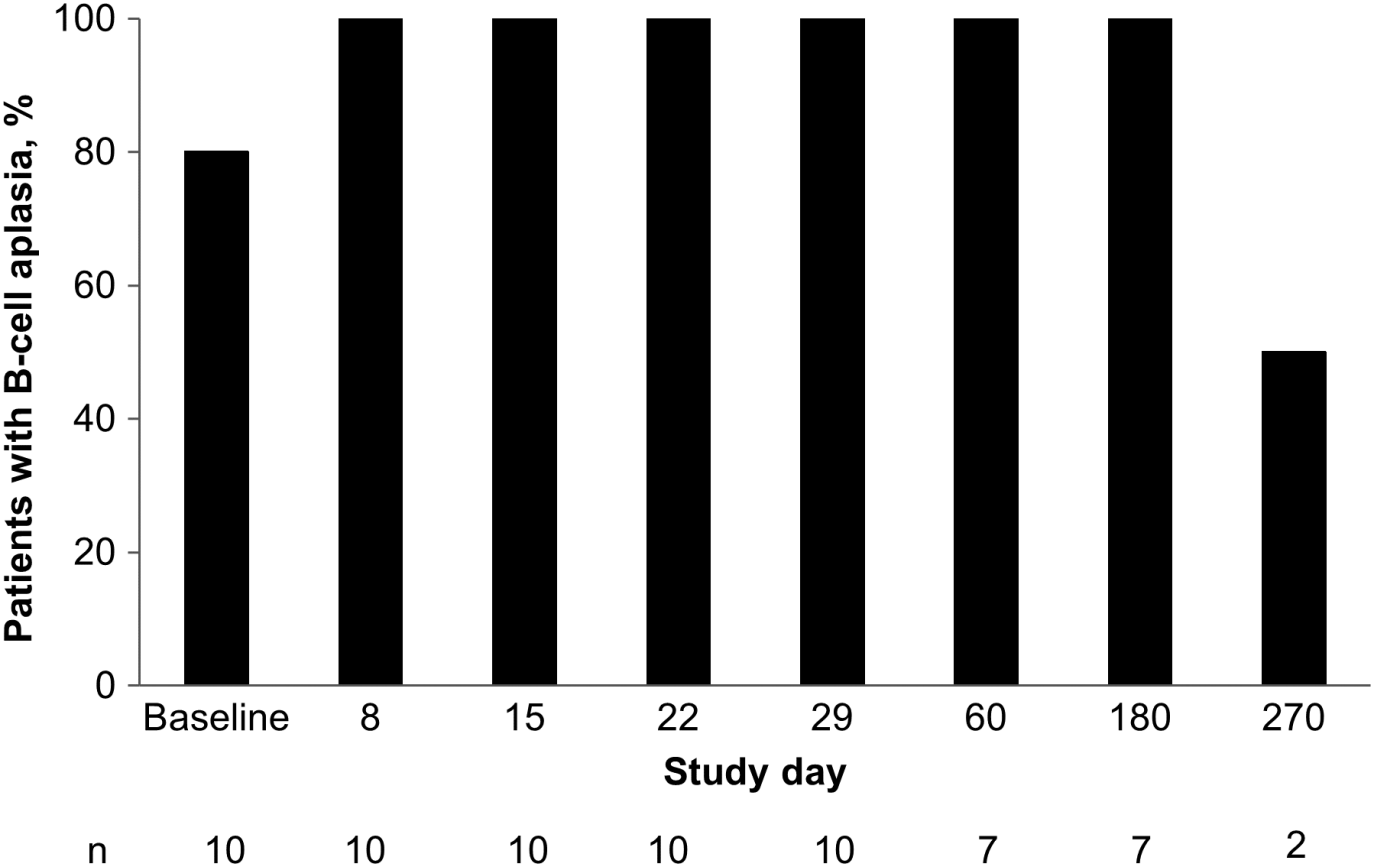
B‐cell aplasia incidence. B‐cell aplasia, defined as CD19^+^ B cells <3% of peripheral blood lymphocytes. Baseline was defined as the last non‐missing measurement before liso‐cel infusion, unless otherwise specified.

B‐cell aplasia was evident at baseline in 80% (*n* = 8/10) of patients. Post‐infusion B‐cell aplasia was reported in 100% of evaluable patients from study days 8 to 180 and in 50% of evaluable patients (*n* = 1/2) by day 270 (Figure 4).

### 3.6 Patients who received nonconforming CAR T‐cell product

The 2 patients who received nonconforming CAR T‐cell product both experienced neutropenia, anemia, leukopenia, and thrombocytopenia. One of these patients also experienced grade 2 diarrhea, grade 2 CRS, and grade 2 hypogammaglobulinemia; this patient achieved a CR, was alive at the data cutoff date, and had a PFS of 12.5 months from product infusion. The other patient experienced grade 1 vomiting and grade 1 fever, then later disease progression (PFS of 1 month from product infusion); the patient died about 2 months later.

### 3.7 Patient narrative: secondary CNS lymphoma

**FIGURE 5:**
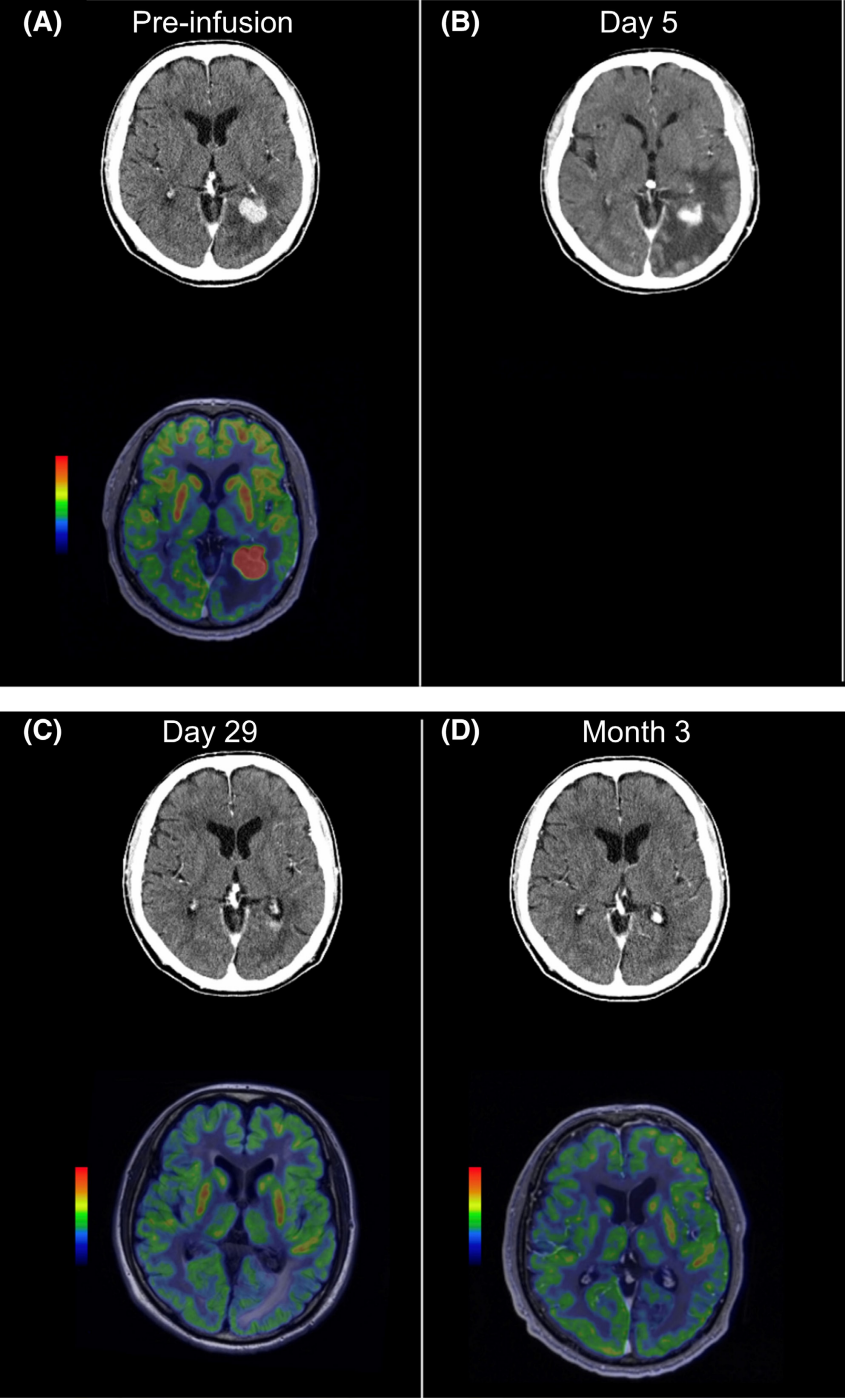
Patient with secondary CNS lymphoma. (A) The patient received two cycles of high‐dose methotrexate to prevent rapid progression of the brain mass lesion, with a response of stable disease (pre‐infusion). (B) A computed tomography scan on day 5 showed decrease of the CNS lesion while peritumoral cerebral edema was observed. (C) To improve the cerebral edema, high‐dose steroids (dexamethasone 20 mg/day every 6 h for 2 days and tapered) were started. After the initiation of steroid, the patient's symptoms rapidly improved, and he achieved a complete metabolic response per investigator assessment on day 29. (D) The complete metabolic response persisted for 3 months after infusion (day 102). CNS, central nervous system.

One 53‐year‐old male patient with primary testicular DLBCL was diagnosed with secondary CNS lymphoma after screening but before liso‐cel infusion. The patient had received three prior lines of therapy but relapsed with subcutaneous lesions within 2 months of the last line of therapy, as seen on gadolinium‐enhanced MRI. After leukapheresis, the patient received gemcitabine as a bridging therapy that was effective in treating the subcutaneous lesions. Although the patient had no neurological or other symptoms, a brain parenchymal lesion was detected by contrast‐enhanced CT scan during the pretreatment evaluation. Meningeal infiltration was not suspected, with negative cerebrospinal fluid. Subsequently, the patient received two cycles of high‐dose methotrexate to prevent rapid progression of the brain mass lesion, with a response of stable disease (Figure 5A). Because he remained neurologically asymptomatic, the patient was considered eligible for treatment per protocol and the decision was made to proceed with liso‐cel infusion.

Two days after infusion, the patient experienced grade 2 CRS, with symptoms of fever (temperature, 38.7°C) and hypotension (74/43 mm Hg) that responded to tocilizumab and hydration. The patient also experienced grade 1 confusion and nausea on day 5. A CT scan showed a reduction of the CNS lesion; peritumoral cerebral edema was also observed, which may have been associated with an antitumor immune response (Figure 5B). Dexamethasone (20 mg/day every 6 h for 2 days and tapered) was administered to treat the cerebral edema. The patient experienced a rapid improvement in symptoms after the initiation of high‐dose steroids and achieved a complete metabolic response by PET scan on day 29 (Figure 5C), which persisted for 3 months after infusion (Figure 5D). Despite exposure to high‐dose steroids, liso‐cel remained detectable 6 months after infusion. The patient achieved a CR per investigator assessment on days 29, 90, and 180, and progressive disease on day 270. Per IRC, the patient achieved a PR on days 29 and 90, a CR on day 180, and progressive disease on day 270.

## 4 DISCUSSION

Cohort 3 of TRANSCEND WORLD is the first study to evaluate the clinical safety and efficacy profile of liso‐cel in Japanese patients with R/R LBCL and is representative of most patients diagnosed with DLBCL in Japan with respect to histology, age, and general disease burden. This study allowed patients with secondary CNS lymphoma to be included, as well as bridging therapy while CAR T cells were being manufactured. After a 12.5‐month median follow‐up, rapid and durable efficacy, with a manageable safety profile, was demonstrated in this patient cohort. The ORR was 70% by IRC and median OS was nearly 15 months. Notably, CRS and NEs occurred at low rates, with no events reported as grade ≥ 3, and all events resolved rapidly. Most TEAEs were cytopenias, which was expected given the prior treatment and LDC. Infection and hypogammaglobulinemia rates were low.

Safety and efficacy outcomes in Cohort 3 were generally similar to those observed in the pivotal TRANSCEND NHL 001 LBCL study (N = 269) conducted in the United States.^6^ While direct comparison of outcomes is limited by the small sample size of TRANSCEND WORLD Cohort 3, naive comparisons indicated comparable ORR and CR rates (~70% and ~ 50%, respectively) for the two populations.^6^ Rates of all‐grade CRS and NEs in Cohort 3, all of which were low grade, were also similar to the results from TRANSCEND NHL 001.^6^

One patient in this cohort was diagnosed with secondary CNS lymphoma after screening but before liso‐cel infusion and achieved a complete durable response. In the pivotal TRANSCEND NHL 001 study, there were six efficacy‐evaluable patients with secondary CNS involvement, three of whom achieved a CR; none had severe CRS, and two patients experienced grade 3 NEs.^6^ Hypofibrinogenemia, which has been associated with CD19‐directed CAR T‐cell therapy in patients with LBCL,^28^, ^29^, ^30^ was observed at a higher rate in Cohort 3 than TRANSCEND NHL 001.^6^ However, no severe bleeding events were reported as a result of liso‐cel treatment in this patient population.

Two patients experienced relapse after achieving a CR after liso‐cel infusion. Because information on biopsy at relapse after liso‐cel was not available at data cutoff, mechanisms of resistance in these patients are unclear. Mechanisms of relapse after CR with CAR T‐cell therapy is not entirely understood and is an active area of interest. Hypotheses include exhaustion of CAR T cells, changes to the tumor microenvironment, and loss or modulation of CD19 target antigen. However, in a post hoc analysis of patients who had prior CD19‐directed therapies before receiving liso‐cel in TRANSCEND NHL 001, 92% achieved responses with no apparent effects on cellular kinetics.^31^ Additionally, a study of genomic mechanisms involved in resistance to CAR T‐cell therapy in patients with LBCL found that neither reduced expression nor genomic alteration of CD19 was associated with poor outcomes.^32^

Cellular kinetics and B‐cell aplasia incidence in this cohort were also similar to those in the pivotal TRANSCEND NHL 001 study.^6^ No immunologic variables are expected to lead to a difference in product behavior between ethnicities, nor is the CD8^+^:CD4^+^ T‐cell ratio expected to have a different impact in this population. There were no dose‐limiting toxicities in this study, and efficacy and safety outcomes in Cohort 3 were aligned with TRANSCEND NHL 001. Although some drugs have demonstrated racial/ethnic differences in pharmacokinetics,^33^, ^34^ no baseline intrinsic and disease factors have yet been identified that may impact CAR T‐cell expansion or persistence among different ethnic groups. In the TRANSCEND NHL 001 study, higher liso‐cel expansion was associated with response (CR or PR), but there were no apparent differences in cellular kinetics observed between responders and nonresponders in this cohort, likely due in part to the high variability of cellular kinetics and the limited number of patients. However, liso‐cel showed long‐term persistence. Preclinical evidence suggests this may be a benefit of 4‐1BB‐based CAR T‐cell therapy.^23^, ^35^

Three second‐generation anti‐CD19 CAR T‐cell therapies have demonstrated efficacy and safety in Japan for patients with R/R aggressive B‐cell NHL: tisagenlecleucel,^10^ axicabtagene ciloleucel,^9^ and, with this report, liso‐cel. Other CAR T‐cell therapies did not permit enrollment of patients with secondary CNS lymphoma in their respective pivotal trials, but independent, retrospective, investigator‐led studies have demonstrated that these patients can be successfully treated with CAR T‐cell therapy.^11^, ^15^, ^36^, ^37^, ^38^ Based on these results, CAR T‐cell therapies, including liso‐cel, may represent a treatment option in selected patients with secondary CNS lymphoma. However, further evaluation is required to determine the exact role of CAR T‐cell therapy in patients with CNS involvement. In addition, careful toxicity management, including brain edema, might be essential.

While other CAR T‐cell therapies have been studied in Japanese patients, randomized trials comparing all three approved anti‐CD19 CAR T‐cell products are lacking. Actual differences in the safety and efficacy profiles of these products, therefore, remain unclear but may include differences in the CAR T‐cell construct, manufacturing, and/or final infused CAR T‐cell product. The Japanese subgroup analysis of tisagenlecleucel reported an ORR of 78%, grade 3 or 4 CRS per the Penn grading system in two of nine patients (22%), and grade 3 NEs in one of nine patients (11%).^10^ In a study of axicabtagene ciloleucel in Japanese patients reporting an ORR of 87%, grade 4 CRS per the Lee 2014 criteria occurred in one patient (6%); no NEs were reported.^9^

Despite the small number of patients and inherent limitations of the open‐label, single‐arm study design, it is encouraging that the results reported here align with the efficacy results observed in the much larger TRANSCEND NHL 001 study.^6^ Of note, our study enrolled a similar number of patients as were reported for other CD‐19‐directed CAR T‐cell treatments in Japanese patients with R/R LBCL, with a longer follow‐up time of 12.5 months (15 evaluable patients in the study of axicabtagene ciloleucel had a median follow‐up time of 5.5 months^9^ and 9 infused patients for tisagenlecleucel had a median follow‐up duration of 5.3 months for OS).^10^

In conclusion, the majority of Japanese patients with R/R LBCL treated with liso‐cel achieved a response by day 29, with a manageable safety profile. The IRC‐assessed ORR was 70%. In addition, 1 patient with secondary CNS lymphoma achieved a PR after liso‐cel infusion per IRC that later deepened to a CR. CRS and NEs occurred at low rates, with no grade ≥ 3 events reported, and all events resolved. These data suggest that liso‐cel appears to be a reasonable treatment option in Japanese patients with R/R LBCL.

## AUTHORS' CONTRIBUTIONS

Shinichi Makita: Data acquisition and data interpretation; Go Yamamoto: Data acquisition and data interpretation; Dai Maruyama: Data acquisition and data interpretation; Yuki Asano‐Mori: Data acquisition and data interpretation; Daisuke Kaji: Data acquisition and data interpretation; Revathi Ananthakrishnan: Study conception or design, data analysis, and data interpretation; Ken Ogasawara: Data analysis, and data interpretation; Lara Stepan: Data analysis, and data interpretation; Claudia Schusterbauer: Study conception or design, data analysis, and data interpretation; Nils Rettby: Study conception or design, data analysis, and data interpretation; Jens Hasskarl: Study conception or design, data analysis, and data interpretation; Koji Izutsu: Data acquisition and data interpretation.

## CONFLICT OF INTEREST

Shinichi Makita has received honoraria from Celgene, a Bristol‐Myers Squibb Company, Chugai, Daiichi Sankyo, Eisai, Novartis, and Takeda. Go Yamamoto, Dai Maruyama, Yuki Asano‐Mori, and Daisuke Kaji have no conflicts to report. Revathi Ananthakrishnan, Ken Ogasawara, and Lara Stepan are employees of Bristol Myers Squibb and may own stock in Bristol Myers Squibb. Jens Hasskarl was an employee of Celgene, a Bristol‐Myers Squibb Company, at the time of this analysis and may own stock in Bristol Myers Squibb. Nils Rettby and Claudia Schusterbauer are employees of Celgene, a Bristol‐Myers Squibb Company, and may own stock in Bristol Myers Squibb. Koji Izutsu has received research funding from Celgene, a Bristol‐Myers Squibb Company.

## ETHICS STATEMENT

The study adhered to the ethical principles of the Declaration of Helsinki and Good Clinical Practice guidelines. Written informed consent was obtained from each patient. An independent ethics committee at each site reviewed and approved the protocol before initiating the study.

## Supporting information


Appendix S1

## Data Availability

Bristol Myers Squibb policy on data sharing may be found at https://www.bms.com/researchers‐and‐partners/independent‐research/data‐sharing‐request‐process.html.
