# Supplementary material for: Phase 2 results of lisocabtagene maraleucel in Japanese patients with relapsed/refractory aggressive B‐cell non‐Hodgkin lymphoma
Source: Cancer Med. 2022 May 26;11(24):4889–99. doi: 10.1002/cam4.4820 (PMC9761090; doi:10.1002/cam4.4820)
Supplement: Supplementary file 1 — Appendix S1 [file CAM4-11-4889-s001.docx]

**Supplementary Appendix**

**Phase 2 results of lisocabtagene maraleucel in Japanese patients with relapsed/refractory aggressive B-cell non-Hodgkin lymphoma**

Shinichi Makita, Go Yamamoto, Dai Maruyama, et al

**Table of Contents**

[METHODS 3](#_Toc77926200)

[Study design 3](#_Toc77926201)

[Cytokine release syndrome (CRS) and neurological events (NE) management protocol 3](#_Toc77926202)

[RESULTS 4](#_Toc77926203)

[FIGURE S1. Platelet count (observed and change from baseline [PRE]^a^) in Cohort 3 of liso-cel–treated patients with a complete or partial response, 10^9^/L 4](#_Toc77926204)

[Figure S2. Cellular kinetic parameters by response. 6](#_Toc77926205)

[Table S1. Anticancer (bridging) therapy for disease control 7](#_Toc77926206)

METHODS

Study design

TRANSCEND WORLD is a single-arm, open-label, multinational, multicohort, phase 2 study in adult patients with diffuse large B-cell lymphoma (DLBCL) not otherwise specified (NOS; de novo or transformed follicular lymphoma [tFL]), high-grade B-cell lymphoma (HGBCL) with *MYC* and *BCL2* and/or *BCL6* rearrangements with DLBCL histology, and follicular lymphoma grade 3B (FL3B) (Cohort 1 and Cohort 3); patients with DLBCL who are transplant ineligible (Cohort 2 and Cohort 3); patients with HGBCL with *MYC* and *BCL2* and/or *BCL6* rearrangements with DLBCL histology (Cohort 4); patients with primary central nervous system lymphoma (Cohort 5); and patients meeting eligibility criteria for Cohort 1 and suitable for outpatient treatment (Cohort 7).

Cytokine release syndrome (CRS) and neurological events (NE) management protocol

Patients with grade 1 CRS with onset ≥72 h after liso-cel infusion were monitored and treated symptomatically. However, patients with rapid onset (<72 h after liso-cel infusion) of CRS or rapid progression of CRS symptoms (<72 h from liso-cel infusion) and patients with grade 2 CRS received tocilizumab 8 mg/kg intravenously and dexamethasone 10 mg intravenously every 12–24 h. If CRS did not improve or progressed within 24 h after the patient received the initial dose of tocilizumab, second-line treatment consisted of an additional dose of tocilizumab 8 mg/kg and dexamethasone 20 mg every 6–12 h. If third-line treatment was required due to lack of improvement or rapid progression of CRS, patients were administered methylprednisolone 2 mg/kg followed by 2 mg/kg divided 4 times per day, and alternative anti-IL6 agents were considered. Fourth line treatments included anti-T-cell therapies such as cyclophosphamide.

First-line treatment for NEs included prophylactic anti-seizure medication for patients at high risk of NEs. NEs with an early onset (<72 h from liso-cel infusion) were treated with dexamethasone 10 mg every 8–12 h if grade 1 or 2, or dexamethasone 20 mg every 6–8 h if grade 3 or every 6 h if grade 4. For NEs with a late onset (≥72 h), grade 1 NEs were observed and dexamethasone 10 mg every 12–24 h could be considered for grade 2 NEs. Treatment of grade 3 NEs varied depending on the type of event. Corticosteroids were not administered for isolated grade 3 headache. For patients with aphasia or confusion, dexamethasone 10 mg every 12 h was given. For patients with depressed level of consciousness, dexamethasone 20 mg every 12 h was given. Patients with grade 4 NEs were treated with dexamethasone 10–20 mg every 6–8 h, with higher dose and shorter intervals for events requiring respiratory support or experiencing seizures.

Second- and third-line treatment of NEs comprised increased doses or more frequent administration of dexamethasone or treatment with methylprednisolone. For patients with life-threatening complications (ie, requiring respiratory support of experiencing seizures), methylprednisolone (2 mg loading dose followed by 2 mg/kg divided 4 times daily) was administered. Patients who experienced cerebral edema received methylprednisolone 1–2 g every 24 h, if needed.

RESULTS

FIGURE S1. Platelet count (observed and change from baseline) in Cohort 3 of liso-cel–treated patients with a complete or partial response, 10^9^/L


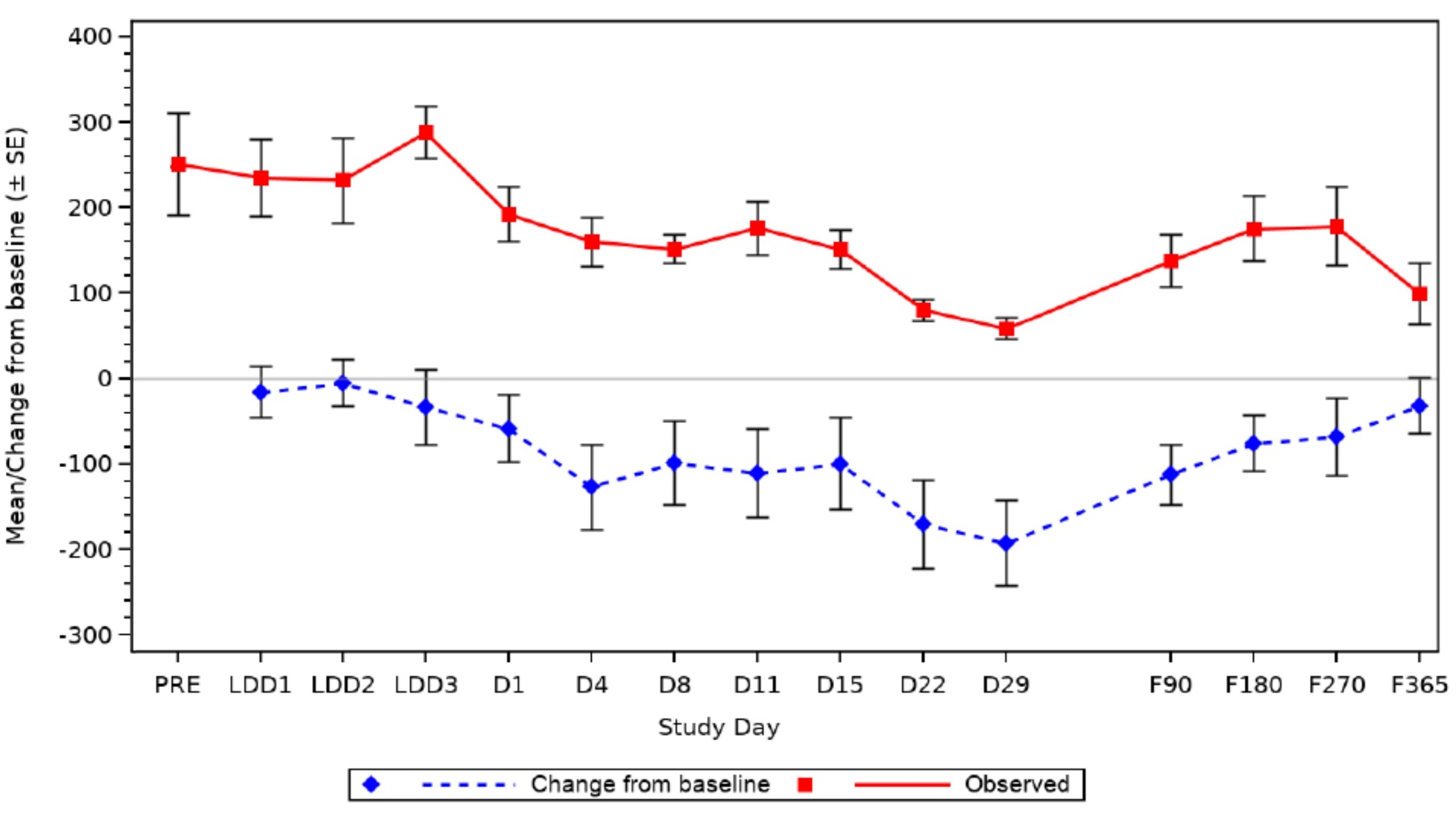


D, study Day; F, follow-up Day; LDD, lymphodepleting chemotherapy Day; LEU, leukapheresis; PRE, pretreatment Day; SCR, screening; SE, standard error.

Figure S2. Cellular kinetic parameters by response


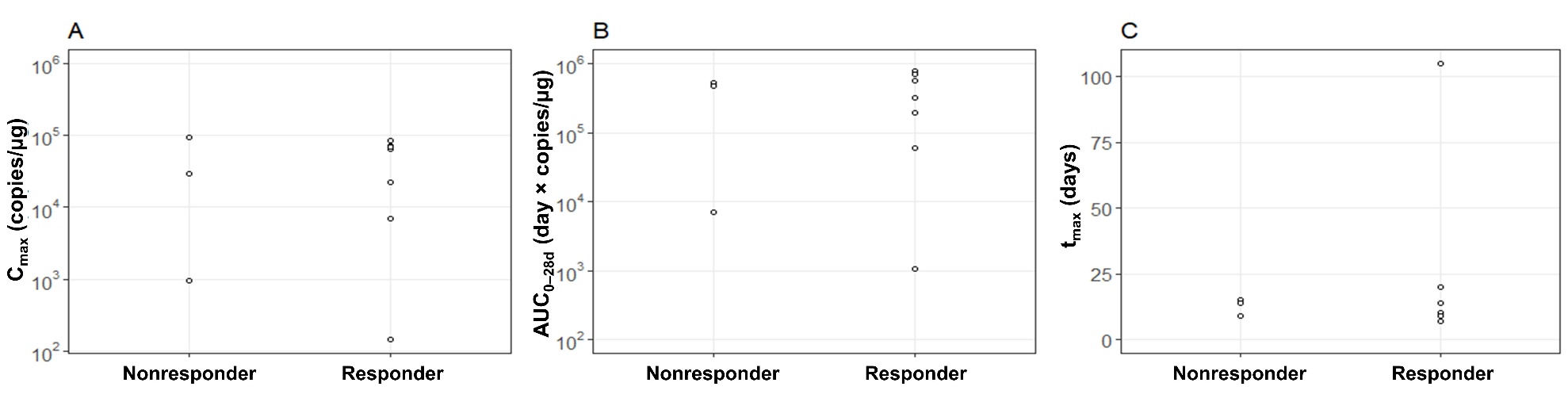


AUC_0–28d_, area under the curve from 0 to 28 days post-infusion; C_max_, maximum expansion; t_max_, time to C_max_.

Table S1. Anticancer (bridging) therapy for disease control

|  | Liso-cel–treated patients (N = 10) |
| --- | --- |

| Received bridging therapy*, n (%) |
| --- |

| Yes | 10 (100) |
| --- | --- |
| No | 0 |
| Type of treatment, n (%) |  |
| Systemic therapy only | 9 (90) |
| Radiotherapy only | 0 |
| Both | 1 (10) |
| **Patient** | **Bridging therapy used** |
| 1 | Carboplatin, etoposide, ifosfamide, rituximab |
| 2^†^ | Cisplatin, cytarabine, etoposide, methylprednisolone, rituximab  Gemcitabine |
| 3 | Gemcitabine, rituximab |
| 4^†^ | Gemcitabine  Methotrexate |
| 5 | Cyclophosphamide, cytarabine, dexamethasone, etoposide, rituximab |
| 6^†^ | Cisplatin, dexamethasone, gemcitabine, rituximab  Dexamethasone |
| 7 | Carboplatin, etoposide, ifosfamide, rituximab |
| 8^†^ | Carboplatin, dexamethasone, etoposide, ifosfamide, rituximab  Dexamethasone |
| 9 | Carboplatin, etoposide, ifosfamide |
| 10 | Carboplatin, dexamethasone, etoposide, ifosfamide, rituximab |

*Anticancer treatment for disease control is defined as any systemic or radiation therapy provided to patients for disease control after leukapheresis and before lymphodepleting chemotherapy.

^†^Patient received more than one regimen of bridging therapy.
